# Is Emissions Trading Scheme (ETS) an Effective Market-Incentivized Environmental Regulation Policy? Evidence from China’s Eight ETS Pilots

**DOI:** 10.3390/ijerph19063177

**Published:** 2022-03-08

**Authors:** Shanglei Chai, Ruixuan Sun, Ke Zhang, Yueting Ding, Wei Wei

**Affiliations:** 1 Business School, Shandong Normal University, Jinan 250358, China; chaishanglei@sdnu.edu.cn (S.C.); 2020020968@stu.sdnu.edu.cn (R.S.); 201922020229@stu.sdnu.edu.cn (K.Z.); 2 School of Management and Economics, Beijing Institute of Technology, Beijing 100081, China; tane2009@126.com; 3 Center for Energy, Environment & Economy Research, School of Management, Zhengzhou University, Zhengzhou 450001, China

**Keywords:** environmental regulation policy, carbon emissions reduction effect, emission trading scheme (ETS), policy evaluation, difference-in-differences (DID), data envelopment analysis (DEA)

## Abstract

Climate change and environmental issues caused by carbon emissions have attracted the attention of governments around the world. Drawing on the experience of the EU, China is actively developing a national carbon emissions trading market, trying to encourage emission entities to incorporate carbon emissions reduction into production and consumption decisions through carbon pricing. Is this scheme an effective market-incentivized environmental regulatory policy? Since China successively launched ETS pilots in 2013, the effectiveness of reducing carbon emissions has become one of the current focus issues. This study uses the difference-in-differences (DID) method to evaluate the impact of ETS implementation on emissions reduction and employs the Super-SBM model in data envelopment analysis (DEA) to evaluate the emission-reduction efficiency of eight ETS pilots in China. We find that the carbon trading policy has achieved emission-reduction effects in the implementation stage, and the greenness of economic growth has a significant positive impact on regional GDP. The establishment of China’s unified carbon market should be coordinated with regional development. Some supporting measures for regional ecological compensation and the mitigation of regional development are yet to be adopted.

## 1. Introduction

Extreme climate change, characterized by warming, poses threats to human health, economic growth, and social development. Environmental pollution has become a major challenge to human economic and social development, hence drawing profound attention to communities. Energy conservation, emission reduction, and climate change have been incorporated into China’s development strategy. China is committed to the national contribution of ecological priority, green development, environmental orientation, and ecological civilization. The Chinese government has actively adopted stronger policy measures, striving to peak carbon dioxide emissions by 2030 and achieve carbon neutrality by 2060.

Since the signing of the “Kyoto Protocol” in 1997, the establishment of a carbon market has been widely regarded as an effective means to control global carbon emissions [1,2]. As a policy tool to control greenhouse gas emissions, the carbon emissions trading mechanism can achieve the goal of reducing emissions while minimizing overall emissions reduction costs through market transactions. Many countries and regions in the world have begun the practice of carbon emissions trading. According to the “International Carbon Action Partnership (ICAP) 2021 Global Carbon Market Progress Report”, 24 carbon emissions trading systems are currently being implemented, covering greenhouse gas emissions accounting for 16% of the global total. The outbreak of COVID-19 in 2020 has shaken the international political landscape, and the impact of COVID-19 has warned us of the weakness of the world’s ability to deal with global crises today, prompting mankind to reflect deeply on the relationship between humans and nature, with more and more countries joining the march toward the goal of carbon neutrality. On 22 September 2020, the Chinese government promised the world a new goal, which is to strive to reach the peak of carbon emissions by 2030 and carbon neutrality by 2060. The goal of carbon neutrality reflects China’s environmental protection responsibility and determination to respond to climate change, which also puts forward higher requirements for China’s economic and social development. Facing the goal of carbon neutrality, effective energy and environmental policy tools are extremely important in controlling CO_2_ emissions and achieving near-zero emissions [3]. Since 2013, China has successively launched eight pilots in Shenzhen, Shanghai, Beijing, Guangdong, Tianjin, Hubei, Chongqing, and Fujian to implement carbon emissions trading policies. On 16 July 2021, the national carbon emissions trading market was officially launched for trading, while the pilot trading market continued to operate at the same time, providing an effective means for achieving the goal of carbon neutrality.

In the critical period of the “14th Five-Year Plan” for carbon peak and carbon neutrality goals, each pilot has local characteristics that are compatible with its economic development and energy-saving and emission-reduction technologies. The different operating conditions of various pilots have resulted in the uneven development of various pilots, which has brought certain obstacles to the establishment of a national carbon market [4]. China’s carbon market has high prices, severe volatility, poor liquidity, and poor information transparency [5]. Measuring the efficiency of the carbon market provides important insights for market participants in formulating trading strategies based on carbon prices [6]. In addition, the efficiency of the carbon market has a significant impact on certain energy issues, such as power generation, energy consumption, and the development of new energy technologies [7]. Therefore, during the development of the national carbon market, an in-depth analysis of the policy effects and operational efficiency of the eight pilot carbon trading markets is of great significance to provide support for the smooth operation of the national carbon emissions trading market.

In response to the above-mentioned problems in China’s carbon market, this study investigates the policy effects of the carbon emissions trading system in terms of both economic functions and emission-reduction effects, and uses the Super-SBM method to study the operational efficiency of the carbon market and examine the operation of the carbon emissions trading system in depth. This study makes the following contributions: First, we use the DID method and the PSM-DID method to study the economic functions and emission-reduction results of the carbon emissions trading system, which not only avoids the heterogeneity caused by the original DID method but also ensures the accuracy of the research results. Second, this study innovatively added the green development index as an indicator to test the emission-reduction effect of the carbon emissions trading mechanism and added the economic growth greenness index to test the economic impact of the carbon emissions trading mechanism in constructing the Super-SBM model. Third, this study fills the gap in the existing literature on the efficiency analysis of the Fujian pilot carbon market, the last emerging market launched in April 2016. The establishment of the Fujian carbon market has a certain special significance. It is developed on the basis of accumulating and absorbing the operating experience of the previous seven pilot markets. It is of great significance to China’s future carbon market integration and national development. Therefore, studying the Fujian carbon market is of certain importance and cannot be ignored.

The rest of the paper is organized as follows: Section 2 briefly reviews the related literature. Section 3 introduces the methodology. The data are described in Section 4. Section 5 shows the results and discussions. Finally, we summarized some conclusions and put forward relevant policy implications in Section 6.

## 2. Literature Review

In response to the problems of uneven pilot development, high and volatile carbon market prices, and poor liquidity and information transparency in China’s carbon market, this paper compares previous research. The existing literature attempts to analyze the impact of the introduction of a carbon emissions trading mechanism in China with respect to many aspects, including the operating efficiency of carbon emissions trading policies and the economic impact and emission-reduction effects of China’s ETSs. Zhang and Zhang conducted experiments on the carbon emissions trading market in the power industry by using a subject-based model and pointed out that carbon trading policies can reduce carbon emissions in China by improving air quality [8]. Qi et al. and Peng et al. used the difference-in-differences (DID) model to compare carbon emissions and economic development between pilot areas and nonpilot areas and concluded that the carbon emissions trading policy significantly reduces carbon emissions in the pilot areas without sacrificing economic development as a price [9,10]. Zhang et al. used the DID model to assess the impact of the emissions trading system on carbon emissions reductions and pointed out that the expanded carbon trading mechanism adopted by China will further control carbon emissions [11]. Liu et al. found that the joint operation of the SO_2_ emissions trading market and the carbon emissions trading market may help achieve the dual goals of increasing potential revenue and reducing pollutant emissions [12]. Gao et al. analyzed the effects of China’s pilot carbon emissions trading policies using provincial-level industry three-dimensional panel data and pointed out that ETSs can promote emissions reduction and play a role by influencing the future carbon emissions expectations of enterprises; however, they may cause carbon emission spillover effects among markets [13]. Zhang et al. empirically found that the implementation of carbon emissions rights trading policies has a significant impact on China’s carbon emissions reduction, but the government’s environmental policies have a lagging effect on the impact of carbon emissions reduction [14]. Yi et al. suggested that the development of the national carbon market should learn from successful pilot experiences in Hubei, Beijing, and Shanghai [15]. Yu et al. proposed that the carbon trading mechanism has effectively improved the carbon performance levels of pilot provinces and cities, and its improvement effect shows regional heterogeneity [16]. Yang et al. conducted a DID analysis on carbon emissions trading pilot policies after controlling important variables such as environmental regulations, population size, and economic level and concluded that the carbon trading mechanism has led to the expansion of employment and the reduction of carbon emissions [17].

The above-mentioned literature widely uses the DID method to analyze the effect of carbon market operation. However, this method cannot solve the endogeneity problem caused by selection bias. To address endogeneity issues, Heckman et al. proposed a propensity score matching and difference-in-differences (PSM-DID) method, which can be used for the experimental group, as each subject matches the best control sample to alleviate the problem of sample selection bias [18]. Based on the above analysis, this paper uses the PSM-DID model to further examine the results of the DID method and verify the environmental and economic impact of the carbon emissions trading policy. In addition, another innovation of this study that differs from previous literature is that we not only use the green development index as an indicator to test the emission-reduction effect of the carbon emissions trading mechanism but also use the economic growth greening degree index to test the economic impact of the carbon emissions trading mechanism. These are two elements that the existing literature ignores, and ignoring these two elements will cause omissions in the analysis of the effects of carbon emissions policies.

Although the above analysis examines the emission-reduction effect and economic impact of the carbon emissions trading mechanism from the overall effect, there is still a research gap regarding the heterogeneity of carbon trading in various regions for the efficiency of China’s carbon market [19]. Therefore, in the current period of a smooth transition from the pilot carbon market to the national carbon market, evaluating the differentiation of the operating efficiency of the eight carbon pilots has become one of the research priorities. To address this issue, a very popular method is data envelopment analysis (DEA), proposed by Charnes et al. [20]. For example, Lu and Lu used a dynamic DEA model to evaluate the interperiod efficiency and execution efficiency of fossil fuel carbon emissions in European Union (EU) countries [21]. Wu et al. used the DEA model that introduced bargaining games to measure the agricultural greenhouse gas emission efficiency of 15 EU member states [22]. Ji et al. used a modified two-stage DEA model to study the impact of the Organization of Petroleum Exporting Countries (OPEC) on the security of oil imports in East Asia [23]. Amowine et al. used a dynamic slack-based measurement (SBM) model to evaluate the dynamic energy efficiency of 25 African economies from 2007 to 2014 [24]. Zhang et al. used an improved DEA model to measure the energy and environmental efficiency of some countries in Central and Western Europe [25]. Sun and Huang innovatively used the slack-based DEA model to analyze the energy structure and energy-saving potential of BRICS countries in the past 30 years, which has important practical significance [26]. Matsumoto et al. employed the DEA method and the global Malmquist–Luenberger index to evaluate the environmental performance of EU countries based on long-term panel data of EU countries [27].

With the general acceptance of this method in recent years, DEA has been used in an increasing number of studies to solve the efficiency problem in China. Wang et al. used the DEA method to estimate the reduction cost savings and GDP loss recovery rate of China’s carbon emissions trading [28]. Based on three carbon trading schemes, Zhang et al. employed a DEA optimization model to evaluate the impact of carbon trading on the economic output of China’s industrial sector and carbon emissions reduction [29]. Cheng and Mu selected the carbon price, price stability, trading activity, quota tightness, market participation, and other evaluation indicators to construct a DEA evaluation model for the market operation efficiency of seven carbon emissions trading pilots in China from 2014 to 2015 [30]. To study the carbon emissions efficiency and carbon emissions reduction potential of 30 provinces in China, Chen et al. constructed a super slack-based measurement (SBM) and zero-sum gain (ZSG)-DEA model using indicators such as labor, capital, GDP, and carbon price [3].

Although the DEA method is widely used in the above literature for efficiency analysis, there are still controversies in the selection of input and output indicators involved in the construction of the DEA model. The above-mentioned studies mainly used indicators such as labor, capital, GDP, and carbon price when using DEA for analysis but ignored two important factors: First, carbon emissions intensity is conducive to reducing carbon emissions and promoting the sustainable development of society as a whole [31]. Second, energy consumption per unit of GDP has a negative impact on the increase in carbon emissions [32]. Based on the above considerations, this study proposes two aspects of innovation in the efficiency analysis: one is to add the rate of reduction of carbon intensity per unit of GDP to evaluate the efficiency of the carbon market; the other is to add a reduction in energy consumption per unit of GDP to measure the efficiency of the carbon market. In this way, the research results are more scientific and accurate.

In summary, there have been many articles on the policy effects of carbon emissions trading in China, and most of the studies have only studied the economic function and emission-reduction function of the ETS using the DID method, and the results obtained using this single method need to be interpreted with caution. This paper successfully adopts the DID method and PSM-DID method to study the economic function and emissions reduction results of the carbon emissions trading system, which not only avoid the heterogeneity caused by using only the double-difference method, but also better ensures the accuracy of the research results. In addition, most of the existing studies on the operational efficiency of carbon markets only use an CO_2_ emission index to measure the operational efficiency of carbon markets without combining it with regional economic development, which is not comprehensive enough. Therefore, this paper adds the carbon intensity reduction rate per unit GDP and the energy consumption reduction index per unit GDP to study the operational efficiency of eight carbon markets more comprehensively and accurately.

## 3. Methodology

### 3.1. DID Model

The DID method, as one of the commonly used policy effect evaluation methods, can help us to observe the changes of the treatment group before and after the policy [33]. It measures the average treatment effect of the treatment group before and after the policy under the premise that the treatment group and the control group meet the parallel trend assumption. To examine whether a policy is effective, we usually compare individual economic differences before and after the policy is implemented and then evaluate the effect of the policy. Since individual economic entities may be simultaneously affected by macroeconomic policies, climate change, and resource dependence, the use of DID methods can control the ex-ante differences between research objects and effectively separate the true results of policies. Before and after China formulated the carbon emissions trading pilot policy, the carbon dioxide emissions of the pilot provinces and cities changed significantly. We compared the development results of China’s carbon market before and after the implementation of the policy to examine the impact of the implementation of the carbon trading policy on economic development and carbon emissions.

In this study, the carbon emissions trading policy is a quasi-natural experiment that began in 2013, and the observed samples are provincial data, including Beijing, Tianjin, Shanghai, Guangdong, Hubei, Chongqing, and Fujian, while Shenzhen is included in Guangdong Province [34]. The model established in this study is given by

(1) $$\begin{array}{c} \ln CE_{it}=\beta_{o}+\delta_{0}treated_{i}+\delta_{1}period_{t}+\delta_{2}treated_{i}\times period_{t}+\beta_{1}\ln PGDP_{it}\\ +\beta_{2}\ln POP_{it}+\beta_{3}ES+\beta_{4}EC+\beta_{5}EI+\beta_{6}G+\delta_{i}+\lambda_{t}+\varepsilon_{it} \end{array}$$

(2) $$\begin{array}{c} \ln Y_{it}={\beta^{\prime }}_{0}+{\delta^{\prime }}_{0}treated_{i}+{\delta^{\prime }}_{1}period_{t}+{\delta^{\prime }}_{2}treated_{i}\times period_{t}+{\beta^{\prime }}_{1}L_{it}\\ +{\beta^{\prime }}_{2}E_{it}+{\beta^{\prime }}_{3}K+{\beta^{\prime }}_{4}GR+\delta_{i}+\lambda_{t}+\varepsilon_{it} \end{array}$$

where *i* represents the province and *t* represents the year. Variables $\ln CE_{it}$ and $\ln Y_{it}$ are the logarithmic values of industrial CO_2_ emissions and logarithmic values of regional GDP for province *i* in year *t*, respectively. Variable *treated* represents the province implementing the carbon trading policy, with values of 1 and 0 for pilot and nonpilot provinces. Variable *period* represents the period of carbon trading policy implementation, with values of 0 and 1 assigned to it before and after the implementation of the carbon trading policy. $\delta_{i}$ is an individual fixed effect. $\lambda_{t}$ is a time-fixed effect. Variable *ε* is the residual, with $\delta_{2}$ and ${\delta^{\prime }}_{2}$ representing the net effect of the policy.

Equation (1) examines the effect of carbon trading policy on industrial CO_2_ emissions in the province, and Equation (2) examines the effect of carbon trading policy on the gross value of the region, which does not satisfy the basic requirement of randomly selecting experimental groups for quasi-natural experiments because the pilot provinces are not randomly selected. Therefore, additional control variables are needed. The control variables are introduced in Equation (1), and we use the GDP per capita of each region to represent the living standard (*lnPGDP*), the natural logarithm of the number of the resident population in each province to represent the population size (*lnPOP*), the share of secondary industry to represent the economic structure (*ES*), the share of coal consumption to energy consumption to represent the energy consumption structure (*EC*), the ratio of total industrial energy consumption to total industrial output to represent the energy intensity (*EI*), and the green development index (*G*), which is an innovation in this paper, can reflect the overall progress of regional ecological civilization construction in a multi-faceted way, and the use of the above variables can effectively measure the impact of a carbon emissions trading policy on carbon dioxide emissions in the province. In Equation (2), the natural logarithm of the number of employed persons at the end of the year in each region is introduced according to the neoclassical economic growth theory to represent labor (*L*), the natural logarithm of the sum of various energy sources consumed by industries and households in a certain period to represent energy consumption (*E*), the net fixed assets to represent capital (*K*), the innovative inclusion of the economic growth greenness (*GR*) variable in this paper can examine the degree of resource consumption and environmental impact of the regional economy in the growth process, and the above variables can effectively measure the impact of the carbon emissions trading policy on the GDP of the region where it is located.

Since the selection of the eight carbon trading pilot provinces and cities is not random, it is easy to cause deviations in the DID method. However, the PSM method can solve this problem. Its theoretical framework is the “counterfactual reasoning model”, which assumes that any causal analysis object has results in both the observed and unobserved cases when the sample is segmented [35]. The matching of the treatment group and the control group in the PSM-DID method can effectively solve the problem of sample selection bias and heterogeneity and ensure that the research results are more reasonable and reliable.

### 3.2. PSM-DID Model

Heckman et al. proposed the PSM method, which can match the best control sample for each object in the experimental group to alleviate the problem of sample selection bias and meet the parallel trend hypothesis [18]. Compared with the DID method, the experimental group and the control group of the PSM-DID method are more comparable, and the experimental results are more robust. This brings the entire policy closer to the state of natural experimentation and makes the estimation results more accurate and credible. This paper studies the impact of carbon emissions trading policies on the emissions and economic development of pilot provinces, especially propensity score matching based on the DID model, which can solve the problem of selection bias, and the results are more robust.

### 3.3. DEA Model

Data envelopment analysis proposed by Charnes et al. is a nonparametric technical efficiency analysis method based on the evaluated object [20]. It uses mathematical programming to solve the relative efficiency of decision making. It has multiple input and multiple output manufacturing units [36]. The DEA method has the characteristics of a wide application range, relatively simple principles, and has advantages when analyzing multiple inputs and multiple outputs, so it has been widely used in many fields [37]. However, the traditional DEA model cannot distinguish between effective decision-making units on the frontier. To solve this problem, this paper uses a super efficiency model for slack-based measures (Super SBM) proposed by Tone [38] to study the efficiency of the carbon market policy. In DEA, the unit or organization being evaluated is called a decision-making unit (DMU), and assuming that there are *n* decision-making units (DMUs) in the entire system, *ρ* represents the efficiency value of the DMU. Each DMU has *m* inputs *x_i_* (*i* = 1, 2..., *m*) and *q* outputs *y_r_* (*r* = 1, 2..., *q*). The DEA model is shown as:

$$\min\rho =\frac{1}{1-\frac{1}{s}\sum_{i=1}^{s}\frac{s_{r}^{+}}{y_{rk}}}$$

(3) $$s.t\{\begin{array}{c} \overset{n}{\underset{j=1,j\neq k}{\sum }}x_{ij}\lambda_{j}\leq x_{ik}\\ \overset{n}{\underset{j=1,j\neq k}{\sum }}y_{rj}\lambda_{j}+s_{r}^{+}\geq y_{rk}\\ \lambda ,\;s^{-},\;s^{+}\geq 0\\ i=1,\cdots ,m;\;r=1,\cdots ,q;\;j=1,\cdots ,n\;\left(j\neq k\right) \end{array}$$

where *x_ij_* and *y_rj_* are the *i*th input and *r*th output of the *j*th project, respectively, and *s* is the slack variable.

## 4. Data

### 4.1. The Sample for DID Model

The study sample is the relevant data required for the DID model, including the economic level, energy consumption, and emissions in 30 provinces of China from 2009 to 2019. The selection of this sample interval is due to two reasons. First, seven domestic carbon emissions trading pilots were launched after the announcement of the National Development and Reform Commission in 2011 on the implementation of carbon emissions trading pilots. The eight carbon trading pilots in Beijing, Tianjin, Shanghai, Hubei, Guangdong, Chongqing, Shenzhen, and Fujian have undergone three phases of research, design, and trial operation. The Shenzhen carbon market trading platform was the first to be launched in June 2013, and in the one-year period between that beginning and the launch of the Chongqing carbon market trading platform in June 2014, China launched seven pilot carbon markets. In addition, the Fujian carbon market started its operation in December 2016, so this paper takes the data from 2009 to 2012 as prepilot and 2013 to 2019 as postpilot. Second, the 2020 statistical yearbook has not been updated so far; therefore, the sample endpoint is 2019. The data come from the 2009–2019 *China Statistical Yearbook*, the 2009–2019 *China Industrial Statistical Yearbook*, the 2009–2019 *China Industrial Economic Statistical Yearbook*, and the CSMAR database.

For Model (1), either using DID to calculate the policy effect or using PSM-DID to match the control group, it is necessary to control for the differences in the economic development levels between provinces, and there is a correlation between the economic development levels of regions and carbon emissions [21]. Therefore, the study uses the natural logarithm of the gross product per capita of each province as the living standard (*lnpgdp*). The population size of each region is an important factor affecting carbon emissions [39], so we choose the natural logarithm of the resident population in each province as the population size (*lnpop*). China’s carbon dioxide emissions mainly originate from industry, and the main body of carbon emissions trading is mainly in the industrial sector. The industrial development of a region directly affects its carbon emissions, so we take the share of secondary industry as the economic structure (*ES*) in this paper. China is the world’s largest energy-consuming country, and coal is the absolute leader in the country’s energy consumption. Coal consumption has a huge impact on carbon emissions. This article uses coal consumption as a percentage to represent the energy consumption structure (*EC*). Energy intensity is one of the most commonly used indicators for comparing the comprehensive energy utilization efficiency of different countries and regions. It reflects the economic benefits of energy utilization. It can also be used to compare the dependence of the economic development of different economies on energy. The total industrial energy consumption is compared with the ratio of the total industrial output value to the energy intensity (*EI*). The green development index highlights the combination of green and development, highlights the comparison of the level and progress of green development in various provinces, and highlights the guiding role of the government in green management. The regional green development index can reflect local carbon emissions. The green development index (*G*) selected in this article was from the China Green Development Index Report (CGDIR) [40]. The carbon emissions (*CE*) data are calculated by the following equation proposed by Xu et al. [41]:

(4) $$CE=\underset{u}{\sum }CO_{2iu}^{t}=\underset{j}{\sum }E_{iu}^{t}\times EF_{u}\times O_{u}$$

where $CO_{2iu}^{t}$ is the carbon dioxide emissions calculated based on fuel *u* in the *t*-th year in area *i*, $E_{iu}^{t}$ is the consumption of fuel *u* in area *i* in year *t*, $EF_{u}$ is the carbon emissions coefficient of fuel *u* (ton CO_2_/TJ), and *O*_u_ is the proportion of fuel *u* being oxidized.

For Model (2), we selected four factors that affect the level of regional economic development. The number of employees in a region can directly affect its economic development. This paper sets the number of employees in each region as the natural logarithm of *L*. Energy consumption will affect the economic development of a region [42]. We define the natural logarithm of energy consumption in each region as *E*. Fixed asset investment is an important means to promote economic development, and we define the net fixed assets of each region as *K*. *GR* is the greenness of economic growth of each region, which is a comprehensive evaluation of the degree of greenness in the process of economic development of a region and is closely related to the level of economic development of the region where it is located.

### 4.2. The Sample for DEA Model

In accordance with the indicators proposed by Zhang et al. for evaluating the operating efficiency of the carbon trading market, this paper reselects and designs the indicator system on this basis [29]. To evaluate the efficiency of China’s carbon trading market in reducing carbon emissions, the panel data of eight carbon pilots from 2017 to 2019 are selected as samples, including Shenzhen, Shanghai, Beijing, Guangdong, Tianjin, Hubei, Chongqing, and Fujian. In the DEA model, “coverage”, “CCER ratio”, and “MRV number” are used as input indicators *x_i_*, and “reduction rate of energy consumption per unit of GDP”, “carbon market transaction volume”, and “reduction rate of carbon intensity per unit of GDP” are used as output indicators *y_r_*. Among the input indicators, the coverage can describe the number of enterprises included in the carbon market for emissions control, which is a good indicator to describe the activity of the carbon market. China Certified Emission Reduction (CCER) is an emissions reduction certificate obtained by enterprises through the implementation of projects to reduce greenhouse gases, and enterprises included in the emission control can use a certain number of CCERs to offset the corresponding amount of carbon emissions, so the difference in the offset ratio also affects the efficiency of the carbon market. The measurement, reporting, and verification (MRV) system is a process for quantifying carbon emissions and the quality assurance of data. Among the output indicators, this paper uses the rate of reduction of energy consumption per unit of GDP and the rate of reduction in carbon intensity per unit of GDP to study the operational efficiency of the eight carbon pilots more comprehensively and accurately, and uses the annual trading volume of carbon pilots to measure the market activity of the eight carbon pilots. The specific descriptions of the input–output indicators are shown in Table 1. The data come from the *China Energy Statistical Yearbook* and the Wind database. To avoid the normalized index value being 0, which is not suitable for the Super-SBM model, this study adopts the minimum-maximum normalization method [37] as follows:

(5) $$Y_{ij}=0.1+0.9\times \frac{\max(X_{ij})-\left(X_{ij}\right)}{\max(X_{ij})-\min(X_{ij})}$$

where *X_ij_* is set as the *j*th index of the *i*th area, and *Y_ij_* is the normalized value of *X_ij_*. This section may be divided by subheadings. It should provide a concise and precise description of the experimental results, their interpretation, as well as the experimental conclusions that can be drawn.

## 5. Empirical Results and Discussions

### 5.1. The Effectiveness Results of Carbon Emissions Trading Policies

**Figure 1:**
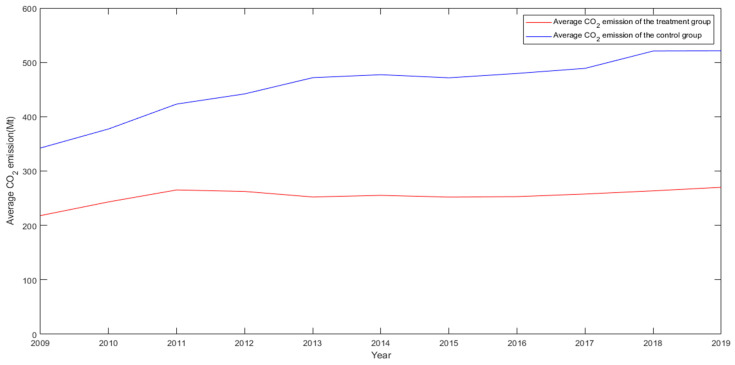
Trend of average CO_2_ emissions from 2009 to 2019.

**Figure 2:**
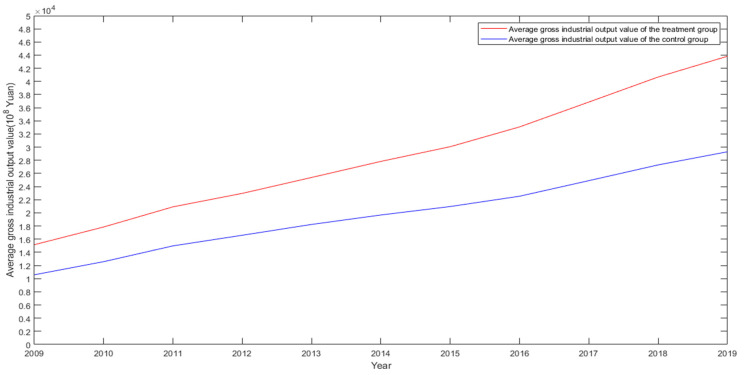
Trend of average GDP from 2009 to 2019.

The premise of the validity of the DID estimation is that it can pass the parallel trend test [43]. The parallel trend test requires that the treatment group and the control group have the same trend before the policy is implemented. If the assumption is not met, then the DID estimate may not be the net effect of the policy but the effect that contains the characteristics of the sample itself. Figure 1 and Figure 2 show the change trend of the average CO_2_ emissions and GDP of the experimental group and the control group from 2009 to 2019. The results all support the validity of the DID method.

#### 5.1.1. Benchmark DID Estimation Results

Table 2 shows the regression results of Equations (1) and (2), using Stata15.1 software. For Equation (1), *lnCE* represents the impact of carbon emissions trading policies on reducing emissions and promoting economic growth. The coefficient of the treated * period term is negative and significant at the 1% level, indicating that the implementation of carbon emissions trading policies can significantly reduce carbon emissions in the pilot areas compared to the nonpilot areas. In addition, population size (*lnPOP*), economic structure (*ES*), energy consumption structure (*EC*), and energy intensity (*EI*) have a significant positive impact on carbon dioxide emissions.

For Equation (2), *lnY* represents the impact of carbon emissions trading policies on promoting economic growth. The coefficient of the treated * period term is significantly positive and significant at the 5% level, indicating that the implementation of the carbon emissions trading policy has a positive effect on economic growth. Economic growth greening (*GR*) has a significant positive impact on GDP growth at the 5% level. The regression results show that the carbon emissions rights trading policy has achieved a significant economic growth effect while reducing carbon dioxide emissions, theoretically proving the effectiveness of the carbon emissions trading policy and the necessity of establishing a national emission trading market.

#### 5.1.2. PSM-DID Estimation Results

The difference-in-differences method is applicable to natural experiments in which the experimental group is randomly generated. It is assumed that the experimental group and the control group are the same except for the experimental variables of the policy shock. However, DID cannot solve the endogeneity problem caused by selection bias. To avoid this issue, this paper chooses the PSM-DID method to estimate the impact of the implementation of the carbon emissions trading policy on carbon emissions and economic growth, and the results are shown in Table 3.

Consistent with the benchmark DID results, the coefficient of the treated * period term in Equation (1) is significantly negative at a level of 10%, indicating that the carbon emissions trading policy can significantly reduce carbon dioxide emissions in the pilot areas in the assessment of a more rigorous measurement model. For Equation (2), the coefficient of the treated * period term is significantly positive at the 5% level, indicating that the implementation of carbon emissions trading policies can significantly increase the GDP of the pilot areas and promote local economic growth. Therefore, we can conclude that the implementation of the carbon emissions trading policy can not only reduce the carbon dioxide emissions in the area where the policy is implemented but also improve the local economic development level.

### 5.2. The Results of Carbon Market Efficiency

The efficiency coefficients, comprehensive technical efficiency (CRSTE), pure technical efficiency (VRSTE), scale efficiency (SCALE), and returns to SCALE of China’s eight carbon trading markets are calculated by MaxDEA Ultra software, and the results are shown in Table 4.

**Figure 3:**
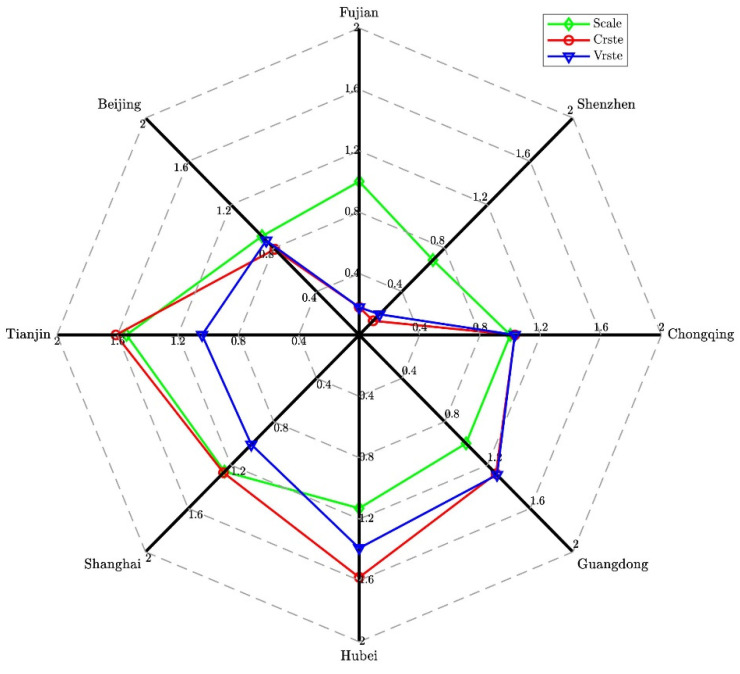
DEA efficiency measurement of the carbon market in 2017.

**Figure 4:**
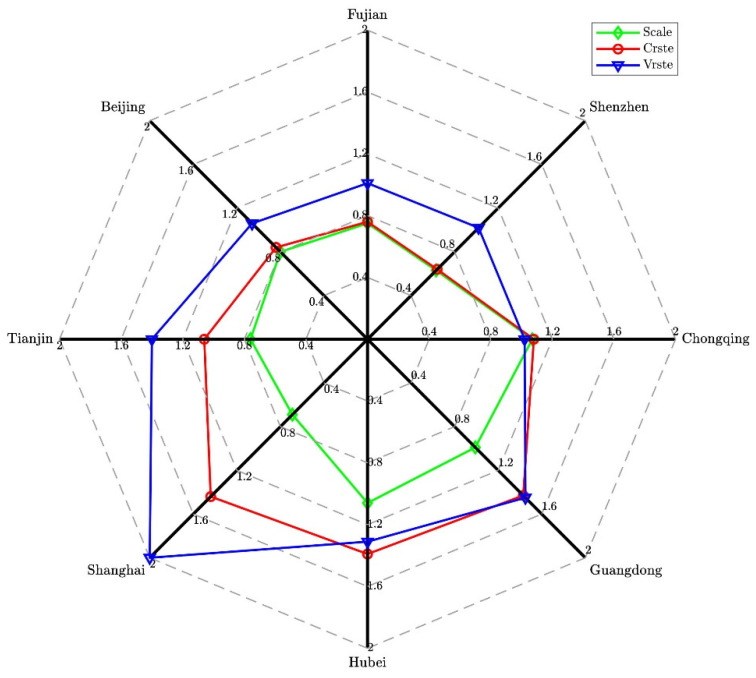
DEA efficiency measurement of the carbon market in 2018.

**Figure 5:**
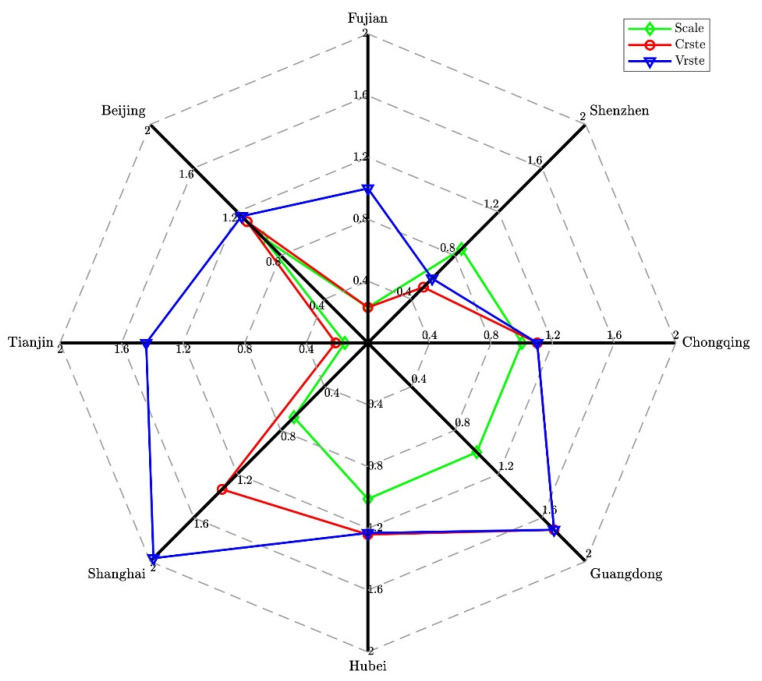
DEA efficiency measurement of the carbon market in 2019.

Furthermore, a year-by-year analysis of the emissions reduction efficiency of China’s carbon trading market from 2017 to 2019 is shown in Figure 3, Figure 4 and Figure 5. The SCALE efficiency values of Tianjin, Shanghai, Hubei, Guangdong, Chongqing, and Fujian in 2017 were all greater than or equal to one, indicating that the emission-reduction efficiency of the carbon trading market was effective in the above-mentioned carbon pilots (see Figure 3). However, the efficiency values of Beijing and Shenzhen did not achieve satisfactory results, and the SCALE efficiency of Shenzhen showed a downward trend in 2017. These two pilots may have extensive production methods that rely on expanding inputs of production factors to increase economic output. Therefore, the Beijing and Shenzhen pilots can optimize the operating efficiency of the carbon trading market by increasing output or reducing input factors.

In 2018, only the Hubei and Chongqing carbon pilots had a SCALE efficiency value greater than one, indicating that these pilots achieved the effect of carbon emissions reduction through the carbon trading market (see Figure 4). The SCALE efficiency of the other carbon pilots was less than one in 2018, which shows that the carbon emissions reduction effect achieved through the carbon trading market is not satisfactory. However, the pure technical efficiency values of the eight carbon trading markets were all greater than one, indicating that they may all pay attention to investment in technology. Carbon trading pilots whose SCALE efficiency did not reach one could improve SCALE efficiency by expanding the scale.

In 2019, the SCALE efficiency values of Hubei, Guangdong, and Chongqing were all greater than or equal to one, indicating that these pilots achieved the effect of carbon emissions reduction through the carbon trading market (see Figure 5). The SCALE efficiency values of the remaining five carbon pilots were all less than one, failing to achieve the target emissions reduction effect through the carbon trading market. In addition, it can be seen from the analysis of the operational efficiency of the carbon trading market in the three years from 2017 to 2019 that the carbon trading market in Hubei Province for the three years is greater than one, which is DEA effective.

We summarize the above results and discuss them as follows:

(1) The CRSTE values for Beijing, Guangdong, and Chongqing all gradually increased from 2017 to 2019. Although the CRSTE values for Shanghai and Hubei did not increase year by year, they were all greater than one in these three years. Moreover, the CRSTE values for Shanghai and Hubei in the three years from 2017 to 2019 are both at the forefront of the eight carbon pilots, indicating that the carbon trading markets in Shanghai and Hubei are relatively mature and efficient;
(2) Although the CRSTE of the two carbon trading markets in Beijing and Chongqing have been increasing year by year, their CRSTE values are lower than that for Guangdong, indicating that the Guangdong carbon trading market has been relatively mature in recent years and has higher carbon emissions reduction efficiency, while Beijing and Chongqing carbon trading pilots need to be further improved;
(3) The CRSTE value for the Tianjin carbon trading market in 2017 and 2018 was relatively high, but its CRSTE value in 2019 was the lowest among the eight carbon pilots. In contrast, its VRSTE is continuously increasing, indicating that technology investment is too rigid. The operating efficiency of the Tianjin carbon pilot showed a downward trend from 2017 to 2019 and a significant decline in 2019, which may be mainly due to two aspects. First, the Tianjin carbon market has low market activity, an obvious concentration of transaction volume, and an excessively strong administrative nature. In addition, Tianjin’s carbon market policy is relatively loose, the compliance mechanism lacks effective restraints, and the punishment for noncompliance behaviors of the included enterprises is weak.

**Figure 6:**
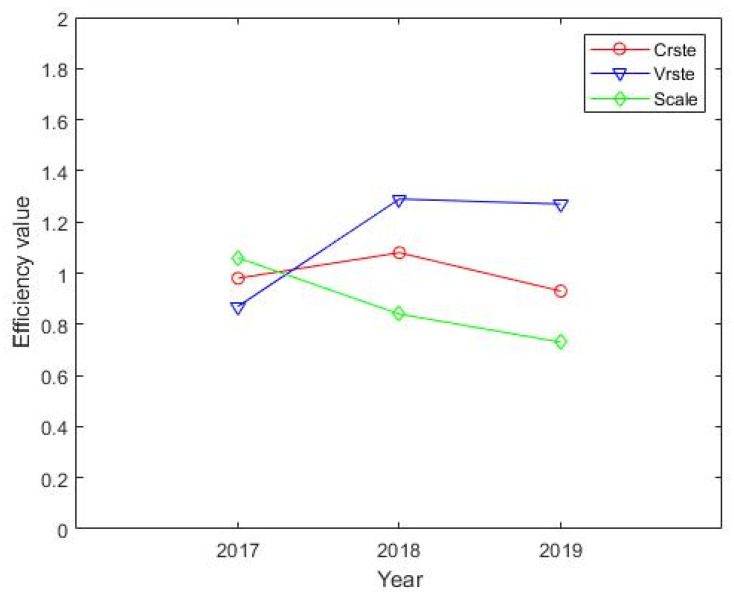
Efficiency and decomposition of China’s carbon trading market.

Furthermore, we used the average method to comprehensively evaluate China’s carbon trading market from 2017 to 2019, and averaged the CRSTE, VRSTE, and SCALE values of eight carbon pilots from 2017 to 2019 year by year and analyzed. The analysis is as follows. Figure 6 shows that the CRSTE and VRSTE for the carbon trading market have basically the same changing trends, both rising first and then falling, and have the same inflection point. In general, the value of VRSTE is greater than the value of CRSTE, and the value of VRSTE also has a greater impact on the value of CRSTE. Generally, technical efficiency is an important factor that affects the operating efficiency of the carbon emissions management system.

### 5.3. Comparison between China’s Carbon Emissions Trading Markets and the European Union Emission Trading Scheme (EU ETS)

As the world’s first and largest carbon emissions trading system, the EU ETS has accumulated some experience in building a carbon market. The EU ETS was put into trial operation in early 2005 and officially put into operation in early 2008. The phased operation mechanism was innovatively adopted for the first time. Based on the Directive 2003/87/EC issued in 2003 and coordinated with a series of financial supervision laws, a relatively perfect legal system was established [44]. The EU ETS is an earlier and more mature organization in the world of carbon trading. Its MRV design framework consists of five parts: monitoring, reporting, verification, quality control, and an exemption mechanism. So far, the EU ETS is one of the most mature carbon markets in the world. Some scholars have studied the impact of the EU ETS on the economy. Martin et al. pointed out that the EU ETS can significantly reduce carbon emissions, and they do not support the view that the existence of a carbon market is not conducive to economic development [45]. Oestriich and Tsiakas pointed out that the performances of companies receiving carbon emissions allowances was significantly better than that of companies without carbon emissions quotas [46].

Although China’s carbon trading pilot has achieved great development, there is still much room for improvement compared with the mature EU ETS. Since 2013, China has successively carried out eight carbon pilots, and online trading was carried out in the national carbon emissions trading market on 16 July 2021. Based on the development characteristics of each region, the first batch of pilot areas have established carbon emissions rights exchanges, established basic trading systems, and continuously reformed and innovated in the pilot process, expanded trading varieties, and created and improved various relevant systems. According to the Measures for the Administration of Carbon Emissions Trading (Trial), the responsibility for the determination and allocation of the total amount of national carbon emissions quotas lie with the Ministry of Ecological Environment and the provincial competent department of the ecological environment. Compared with the EU ETS, the legislative level of China’s carbon market-related policies needs to be improved, and the development of China’s MRV system is not perfect, the data transparency is not high, and there is a lack of control and supervision over the transaction process.

## 6. Conclusions

In the context of global warming in the future, China will take more and more responsibility for energy conservation and emissions reduction, and the implementation of a carbon emissions trading policy is an important means to cope with climate change and ensure sustainable economic and social development. In response to a series of problems such as the unbalanced development of China’s carbon market and the fact that most of the current studies on China’s carbon market are not comprehensive, this study takes China’s carbon emissions trading market as the research object and conducts experiments based on the actual operation of China’s eight carbon trading markets. The DID method was used to test the impact of the implementation of the carbon emissions trading policy on carbon dioxide emissions and economic development, and the PSM-DID method was used to further verify the experimental results of the DID method. The Super-SBM method was used to test the operating efficiency and emission reduction effect of China’s carbon trading market. The main conclusions of the efficiency and emission-reduction effects are as follows: First, the implementation of a carbon emissions trading policy can not only reduce the CO_2_ emissions in the places where the policy is implemented but also promote the economic development of the pilot regions. For the control variables, population size, economic structure, energy consumption structure, and energy intensity have significant positive effects on carbon dioxide emissions, and the greenness of economic growth has a significant positive effect on regional GDP. This indicates that the government should control the population size, adjust the proportion of secondary industry in the GDP, and reduce the proportion of coal consumption in energy consumption and energy consumption per unit of output value, and localities should pay attention to the degree of greenness in the process of economic development and improve the degree of greenness of economic growth. Second, after studying the operational efficiency of China’s eight carbon pilots from 2017 to 2019, this paper evaluates the operational efficiency of each region as follows: Hubei, Guangdong, and Chongqing carbon pilots are outstanding; Beijing, Shanghai, and Shenzhen carbon pilots are in the middle level; and Tianjin and Fujian carbon pilots have significantly lower and more variable operational efficiency values in 2019. This is mainly attributed to the following: the Tianjin carbon market policy is more lenient, the penalty force for the noncompliance of incorporated enterprises is weaker compared to other pilot carbon pilots, and the Tianjin carbon market legislation is only a departmental document, which is less binding. The Fujian carbon market did not resume trading in 2019 until March and had a low level of market activity. Based on the above research conclusions, we draw some policy implications.

China’s carbon emissions trading market was launched online on 16 July 2021 and is still in its initial stage. The establishment of China’s unified carbon market should be coordinated with China’s regional development strategies and development goals so that the national carbon market can contribute to regional ecological compensation and alleviate regional development imbalances. We suggest that less developed regions should be compensated to a certain extent. First, the government can increase the total amount of carbon emissions quotas for less developed regions and give them more space for quota allocation. Second, the government should establish policy measures to encourage the supply of offset mechanism allowances in less developed regions, give priority to offset mechanism projects in less developed regions in the setting of offset mechanisms in the carbon market, and strengthen policy support for energy-saving and emission-reduction projects and carbon sink projects in less developed regions.

The carbon market is highly dependent on laws and regulations due to its human-driven attributes, and the construction of a healthy and efficient carbon market requires perfect legislation. The improvement of China’s carbon emissions trading rules should adhere to the principle of starting from China’s national conditions and practical experience, building a carbon emissions trading mechanism with Chinese characteristics. At the same time, we can learn from the successful experience of other countries and pay attention to the issue of matching and convergence between the laws and regulations related to carbon trading and related international laws and regulations, taking into account China’s own economic and social development situation.

Compared with the more perfect carbon trading information disclosure system in foreign countries, the existing carbon trading system in China lacks transparency and credit benchmarks, and there are still no unified MRV guidelines at the national level. Enterprises are an important participant in the national carbon market. The government’s establishment of a complete carbon emissions information disclosure system and a fair and equitable trading environment can fully mobilize the enthusiasm of enterprises to participate in the construction of the national carbon market, promote the realization of the green development of enterprises, and accelerate the process of energy conservation and emission reduction in China.

The EU ETS development has matured, largely due to its development of carbon quota futures, options, and other carbon financial products in the early days of its establishment. In the process of pilot work in China, eight pilot regions have successively developed products such as carbon bonds, carbon future, and carbon options, but they cannot meet the carbon asset management needs of emission control companies. In the process of establishing a national carbon emissions trading market, access can be appropriately relaxed, and relevant financial institutions and carbon asset management companies can be encouraged to participate in market transactions, guide the development of carbon financial derivatives, and promote relevant enterprises to participate in carbon trading.

However, there are some shortcomings in the research of this paper. When considering the evaluation of carbon market operation efficiency, this paper should consider the influence of other factors on the carbon market operation efficiency, and other input indicators can be added to observe whether different input indicators will affect the carbon market operation efficiency and establish a more complete evaluation system.

## Figures and Tables

**Table 1:** The efficiency rating indicator system of the carbon trading market.

Type	Indicator Name	Indicator Explanation	Data Source
Input indicator	Controlled coverage	Number of controlled enterprises: X1	The official websites of each carbon trading pilot
	CCERs proportion	The number of CCERs that are allowed to be offset by emission control companies in each carbon trading market: X2	The official websites of each carbon trading pilot
	Number of MRV institutions	Carbon verification agency reserves in the ith year of each carbon trading market: X3	The official websites of each carbon trading pilot
Output indicator	Reduction rate of energy consumption per unit of GDP	Decrease in total energy consumption and emission intensity per unit of GDP: Y1	*China Energy Statistical Yearbook*
	Reduction rate of carbon intensity per unit of GDP	Decrease in carbon dioxide emission intensity per unit of GDP: Y2	*China Energy Statistical Yearbook*
	Market activity	Annual trading volume of each carbon pilot: Y3	Wind Database

**Table 2:** DID regression results.

Variables	*lnCE*_it_	*lnY*
treated * period	−0.11 *** (0.36)	0.08 ** (0.03)
*period*	0.44 ** (0.12)	0.98 *** (0.06)
*lnpgdp*	−0.01 (0.03)	
*lnpop*	1.37 ** (0.55)	
*ES*	1.25 *** (0.30)	
*EC*	0.72 *** (0.08)	
*EI*	0.06 ** (0.03)	
*G*	0.07 (0.11)	
*L*		0.17 (0.17)
*E*		−0.05 (0.03)
*GR*		0.42 ** (0.17)
*K*		−0.00 (0.00)
*_cons*	−7.05 (4.43)	8.23 (1.22)
*Year FE*	Controlled	Controlled
*Firm FE*	Controlled	Controlled
*N*	328	325
*R-squared*	0.67	0.88

Note: *, **, and *** indicate that the estimated coefficients are significant at the levels of 10%, 5%, and 1%, respectively. Numbers in brackets are standard error.

**Table 3:** PSM-DID regression results.

Variables	*lnCE*_it_	*lnY*
treated * period	−0.07 * (0.39)	0.11 ** (0.03)
*period*	0.34 ** (0.12)	0.98 *** (0.04)
*lnpgdp*	−0.00 (0.03)	
*lnpop*	0.19 (0.56)	
*ES*	1.33 *** (0.33)	
*EC*	0.57 ** (0.21)	
*EI*	0.09 * (0.04)	
*G*	0.04 (0.12)	
*L*		0.13 (0.22)
*E*		−0.00 (0.06)
*GR*		0.41 * (0.20)
*K*		0.00 (−0.00)
*_cons*	2.63 (4.61)	8.17 (1.53)
*Year FE*	Controlled	Controlled
*Firm FE*	Controlled	Controlled
*N*	148	221
*R-squared*	0.57	0.25

Note: *, **, and *** indicate that the estimated coefficients are significant at the levels of 10%, 5%, and 1%, respectively. Numbers in brackets are standard error.

**Table 4:** The results of DEA efficiency evaluation.

Year	*DMU*	*CRSTE*	*VRSTE*	*SCALE*
Year 2017	Beijing	0.79	0.87	0.91
	Tianjin	1.61	1.04	1.54
	Shanghai	1.27	1.01	1.26
	Hubei	1.58	1.39	1.13
	Guangdong	1.28	1.29	1.00
	Chongqing	1.03	1.03	1.00
	Shenzhen	0.13	0.19	0.69
	Fujian	0.18	0.18	1.00
	Mean	0.98	0.87	1.06
Year 2018	Beijing	0.84	1.06	0.80
	Tianjin	1.06	1.40	0.76
	Shanghai	1.44	2.07	0.69
	Hubei	1.39	1.31	1.06
	Guangdong	1.43	1.45	0.99
	Chongqing	1.08	1.02	1.07
	Shenzhen	0.64	1.02	0.63
	Fujian	0.76	1.01	0.75
	Mean	1.08	1.29	0.84
Year 2019	Beijing	1.11	1.16	0.96
	Tianjin	0.21	1.44	0.15
	Shanghai	1.34	1.97	0.68
	Hubei	1.24	1.23	1.01
	Guangdong	1.71	1.71	1.00
	Chongqing	1.10	1.10	1.00
	Shenzhen	0.51	0.59	0.86
	Fujian	0.23	1.00	0.23
	Mean	0.93	1.27	0.73

## Data Availability

Not applicable.

## References

[1] Spaargaren G., Mol A.P.J. (2013). Carbon flows, carbon markets, and low-carbon lifestyles: Reflecting on the role of markets in climate governance. Environ. Polit..

[2] Wu Q., Wang M., Tian L. (2020). The market-linkage of the volatility spillover between traditional energy price and carbon price on the realization of carbon value of emission reduction behavior. J. Clean. Prod..

[3] Chen X., Lin B. (2021). Towards carbon neutrality by implementing carbon emissions trading scheme: Policy evaluation in China. Energ. Policy.

[4] Zhang Z. (2015). Carbon emissions trading in China: The evolution from pilots to a nationwide scheme. Clim. Policy.

[5] Zhao X., Jiang G., Nie D., Chen H. (2016). How to improve the market efficiency of carbon trading: A perspective of China. Renew. Sust. Energ. Rev..

[6] Zheng J., Yang M., Ma G., Xu Q., He Y. (2020). Multi-agents-based modeling and simulation for carbon permits trading in China: A regional development perspective. Int. J. Environ. Res. Public Health.

[7] Fan X., Lv X., Yin J., Tian L., Liang J. (2019). Multifractality and market efficiency of carbon emission trading market: Analysis using the multifractal detrended fluctuation technique. Appl. Energ..

[8] Zhang H., Zhang B. (2020). The unintended impact of carbon trading of China's power sector. Energ. Policy.

[9] Qi S., Cheng S., Cui J. (2021). Environmental and economic effects of China's carbon market pilots: Empirical evidence based on a DID model. J. Clean. Prod..

[10] Peng H., Qi S., Cui J. (2021). The environmental and economic effects of the carbon emissions trading scheme in China: The role of alternative allowance allocation. Sustain. Prod. Consump..

[11] Zhang Y., Li S., Luo T., Gao J. (2020). The effect of emission trading policy on carbon emission reduction: Evidence from an integrated study of pilot regions in China. J. Clean. Prod..

[12] Liu X., Wang B., Du M., Zhang N. (2018). Potential economic gains and emissions reduction on carbon emissions trading for China's large-scale thermal power plants. J. Clean. Prod..

[13] Gao Y., Li M., Xue J., Liu Y. (2020). Evaluation of effectiveness of China's carbon emissions trading scheme in carbon mitigation. Energ. Econ..

[14] Zhang Y., Peng Y., Ma C., Shen B. (2017). Can environmental innovation facilitate carbon emissions reduction? Evidence from China. Energ. Policy.

[15] Yi L., Bai N., Yang L., Li Z., Wang F. (2020). Evaluation on the effectiveness of China's pilot carbon market policy. J. Clean. Prod..

[16] Yu X., Chen H., Li Y. (2021). The impact of carbon trading mechanism based on synthetic control method on carbon performance. China Popul. Resour. Environ..

[17] Yang X., Jiang P., Pan Y. (2020). Does China’s carbon emission trading policy have an employment double dividend and a Porter effect?. Energ. Policy.

[18] Heckman J.J., Hidehiko I., Todd P.E. (1997). Matching as an econometric evaluation estimator: Evidence from evaluating a job training programme. Rev. Econ. Stud..

[19] Tan X., Wang X. (2017). The market performance of carbon trading in China: A theoretical framework of structure-conduct-performance. J. Clean. Prod..

[20] Charnes A., Cooper W.W., Rhodes E. (1978). Measuring the efficiency of decision making units. Eur. J. Oper. Res..

[21] Lu C., Lu L. (2019). Evaluating the energy efficiency of European Union countries: The dynamic data envelopment analysis. Energ. Environ..

[22] Wu H., Du S., Liang L., Zhou Y. (2013). A DEA-based approach for fair reduction and reallocation of emission permits. Math. Comput. Model..

[23] Ji Q., Zhang H., Zhang D. (2019). The impact of OPEC on East Asian oil import security: A multidimensional analysis. Energ. Policy.

[24] Amowine N., Ma Z., Li M., Zhou Z., Naminse E.Y., Amowine J. (2020). Measuring dynamic energy efficiency in Africa: A slack-based DEA approach. Energy Sci. Eng..

[25] Zhang J., Patwary A.K., Sun H., Raza M., Taghizadeh-Hesary F., Iram R. (2021). Measuring energy and environmental efficiency interactions towards CO_2_ emissions reduction without slowing economic growth in central and western Europe. J. Environ. Manag..

[26] Sun S., Huang C. (2021). Energy structure evaluation and optimization in BRICS: A dynamic analysis based on a slack based measurement DEA with undesirable outputs. Energy.

[27] Matsumoto K., Makridou G., Doumpos M. (2020). Evaluating environmental performance using data envelopment analysis: The case of European countries. J. Clean. Prod..

[28] Wang K., Wei Y., Huang Z. (2016). Potential gains from carbon emissions trading in China: A DEA based estimation on abatement cost savings. Omega.

[29] Zhang W., Li J., Li G., Guo S. (2020). Emission reduction effect and carbon market efficiency of carbon emissions trading policy in China. Energy.

[30] Cheng Y., Mu D. (2017). Evaluation of the operation efficiency of China's pilot carbon market. Sci. Technol. Manag. Res..

[31] Wang Y., Zheng Y. (2021). Spatial effects of carbon emission intensity and regional development in China. Environ. Sci. Pollut. R..

[32] Zhang Y., Fu Z., Xie Y., Li Z., Liu Y., Hu Q., Guo H. (2021). Multi-objective programming for energy system based on the decomposition of carbon emission driving forces: A case study of Guangdong, China. J. Clean. Prod..

[33] Imbens G.W., Wooldridge J.M. (2009). Recent developments in the econometrics of program evaluation. J. Econ. Lit..

[34] Dong F., Dai Y., Zhang S., Zhang X., Long R. (2019). Can a carbon emission trading scheme generate the Porter effect? Evidence from pilot areas in China. Sci. Total Environ..

[35] Zhou B., Zhang C., Song H., Wang Q. (2019). How does emission trading reduce China's carbon intensity? An exploration using a decomposition and difference-in-differences approach. Sci. Total Environ..

[36] Song M., Zhang L., Liu W., Fisher R. (2013). Bootstrap-DEA analysis of BRICS’ energy efficiency based on small sample data. Appl. Energ..

[37] Yang Y., Guo H., Wang D., Ke X., Li S., Huang S. (2021). Flood vulnerability and resilience assessment in China based on super-efficiency DEA and SBM-DEA methods. J. Hydrol..

[38] Tone K. (2002). A slacks-based measure of super-efficiency in data envelopment analysis. Eur. J. Oper. Res..

[39] Aslam B., Hu J., Hafeez M., Ma D., AlGarni T.S., Saeed M., Abdullah M.A., Hussain S. (2021). Applying environmental Kuznets curve framework to assess the nexus of industry, globalization, and CO_2_ emission. Environ. Technol. Inno..

[40] Han J., Guan C. (2019). China Green Development Index Report 2019: Regional Comparison.

[41] Xu X., Zhao T., Liu N., Kang J. (2014). Changes of energy-related GHG emissions in China: An empirical analysis from sectoral perspective. Appl. Energ..

[42] Saldivia M., Kristjanpoller W., Olson J.E. (2020). Energy consumption and GDP revisited: A new panel data approach with wavelet decomposition. Appl. Energ..

[43] Bertrand M., Duflo E., Mullainathan S. (2004). How much should we trust differences-in-differences estimates?. Q. J. Econ..

[44] Deng M.Z., Zhang W.X. (2019). Recognition and analysis of potential risks in China's carbon emission trading markets. Adv. Clim. Chang. Res..

[45] Martin R., Muuls M., Wagner U.J. (2016). The Impact of the European Union Emissions Trading Scheme on Regulated Firms: What Is the Evidence after Ten Years?. Rev. Environ. Econ. Policy.

[46] Oestreich A.M., Tsiakas I. (2015). Carbon Emissions and Stock Returns: Evidence from the EU Emissions Trading Scheme. J. Bank. Financ..

